# The Role of Dietary Histone Deacetylases (HDACs) Inhibitors in Health and Disease

**DOI:** 10.3390/nu6104273

**Published:** 2014-10-14

**Authors:** Shalome A. Bassett, Matthew P. G. Barnett

**Affiliations:** 1 Food Nutrition & Health Team, Food & Bio-based Products Group, AgResearch Limited, Grasslands Research Centre, Tennent Drive, Palmerston North 4442, New Zealand; E-Mail: matthew.barnett@agresearch.co.nz; 2 Nutrigenomics New Zealand, Private Bag 92019, Auckland 1142, New Zealand

**Keywords:** nutritional epigenetics, histone deacetylase, lysine deacetylase, histone deacetylase inhibitors

## Abstract

Modification of the histone proteins associated with DNA is an important process in the epigenetic regulation of DNA structure and function. There are several known modifications to histones, including methylation, acetylation, and phosphorylation, and a range of factors influence each of these. Histone deacetylases (HDACs) remove the acetyl group from lysine residues within a range of proteins, including transcription factors and histones. Whilst this means that their influence on cellular processes is more complex and far-reaching than histone modifications alone, their predominant function appears to relate to histones; through deacetylation of lysine residues they can influence expression of genes encoded by DNA linked to the histone molecule. HDAC inhibitors in turn regulate the activity of HDACs, and have been widely used as therapeutics in psychiatry and neurology, in which a number of adverse outcomes are associated with aberrant HDAC function. More recently, dietary HDAC inhibitors have been shown to have a regulatory effect similar to that of pharmacological HDAC inhibitors without the possible side-effects. Here, we discuss a number of dietary HDAC inhibitors, and how they may have therapeutic potential in the context of a whole food.

## 1. Introduction

Epigenetic changes (*i.e.*, variance in phenotype or patterns of gene expression without a change in the underlying DNA sequence [1]) are essential for the regulation and expression of genes involved in normal growth and development, and in the maintenance of cellular function. Epigenetic dysregulation is associated with a range of negative health outcomes, such as inflammatory disorders of the intestine and colorectal cancer [2]. Although diet has the potential to mitigate these changes, the epigenetic effect of most food components is currently poorly defined.

Modification of the histone proteins associated with DNA is an important epigenetic process within the concept of the dynamic regulation of DNA structure and function. Mechanisms that influence this process are therefore important, and include methylation, acetylation, and phosphorylation. As the name suggests, histone deacetylases (HDACs) are responsible for the removal of the acetyl group from histones, with resulting ability to influence expression of genes encoded by DNA linked to the histone molecule. However, although their primary role is the modification of histones, HDACs also remove the acetyl group from lysine residues in a range of other proteins including transcription factors, hence their alternative name; lysine deacetylases (KDACs). Consequently, their influence on cellular processes is more complex and far-reaching than histone modifications alone. HDAC inhibitors in turn regulate the activity of HDACs, and have been widely used as therapeutics in psychiatry and neurology, in which some disorders associated with aberrant HDAC function (e.g., Alzheimer’s disease) [3]. HDAC inhibitors have also been shown to reduce colonic inflammation [4], inhibit cell proliferation, and stimulate apoptosis, and are in development as promising anti-cancer drugs [5,6]. More recently, dietary HDAC inhibitors have been shown to have a similar regulatory effect as pharmacological HDAC inhibitors without the possible side-effects. There is therefore a significant and growing interest in the use of these as anti-inflammatory and chemo-preventive compounds.

In this review, we briefly describe the development of the understanding of histones, with particular reference to the growing realization of their role in the dynamic regulation of gene expression. We introduce the various mechanisms by which histones are modified, with particular emphasis on those that influence acetylation, and highlight their importance by providing examples of their roles in health and disease. Finally, we discuss in some depth a number of known dietary HDAC inhibitors, and how they may have therapeutic potential in the context of a whole food, rather than using the more traditional pharmacological approach.

## 2. Histones

### 2.1. A Brief History

As early as 1903, it was reported that chromatin consisted of a nucleic acid component and a protein component. The nature of the protein component was not clear, but there was some evidence to suggest that chromatin “may be a compound with a more complex proteid substance, a histon” [7]. Histones were subsequently referred to as an “interesting class of proteins with marked basic properties”, primarily obtained from sperm cells [8]. In 1946, a report referred to the preparation of a “chromosin”, a desoxyribose nucleoprotein complex, from “a great variety of cells” [9]. This report confirmed that a chromosin was from the nuclear fraction, contained “desoxyribose nucleic acid” and histone and non-histone proteins, and that “histones are present in most animal cells and at least in some plant and bacterial cells.” Their role was initially seen as being a key structural component of chromosomes, acting as a protein “scaffold” on which the DNA is arranged. However, with the advent of more advanced molecular biology techniques it became increasingly clear that, far from acting as a passive scaffold, histones have an important dynamic role in the regulation of DNA function [10].

### 2.2. Modifications

Studies on the various “sub-fractions” of histones established that these were produced by side-chain modifications of amino acid residues, which occurred after the proteins had been synthesized. As early as 1968, histone acetylation had been observed, with the identification of an ε-*N*-acetyl derivative of lysine in histones [11]. However, at this time it was supposed that such modifications were permanent, and were a means by which various types of histones were formed. One of the key shifts in thinking in this area of research is that histone modifications are not permanent, and that the systems which control them, in turn, exert a level of control over the expression of the genetic code.

It is now well established that histone residues can undergo a wide array of modifications. At least eight different types of modification have been characterized with a range of enzymes identified for each: acetylation, methylation, phosphorylation, ubiquitination, sumoylation, ADP-ribosylation, deimination, and proline isomerisation. These are discussed in further detail in Section 3. To date, over 60 different modified residues have been identified on histones either by modification-specific antibodies or mass spectrometry [12]. An extra layer of complexity is that arginine and lysine residues can be mono- or di-methylated (arginine) or mono-, di- or tri-methylated (lysine). It has also become apparent that histone modifications can act in concert with other epigenetic mechanisms such as DNA methylation to exert a further level of control over gene expression [13].

## 3. Histone Modifying Enzymes

Many histone modifications are potentially reversible and are regulated by a range of enzymes that can either add or remove these covalent modifications. These changes influence the degree of interaction between DNA and histone, in turn having profound effects on the ability of that DNA to be transcribed (Table 1). However, not all of these modifications will be on the same histone at the same time. Histone modifications can rapidly change (in minutes), allowing the cell to quickly respond to outside stimuli [12]. In addition, many of the enzymes responsible for modifying histone residues also have a number of non-histone substrates such as transcription factors.

**Figure 1:**
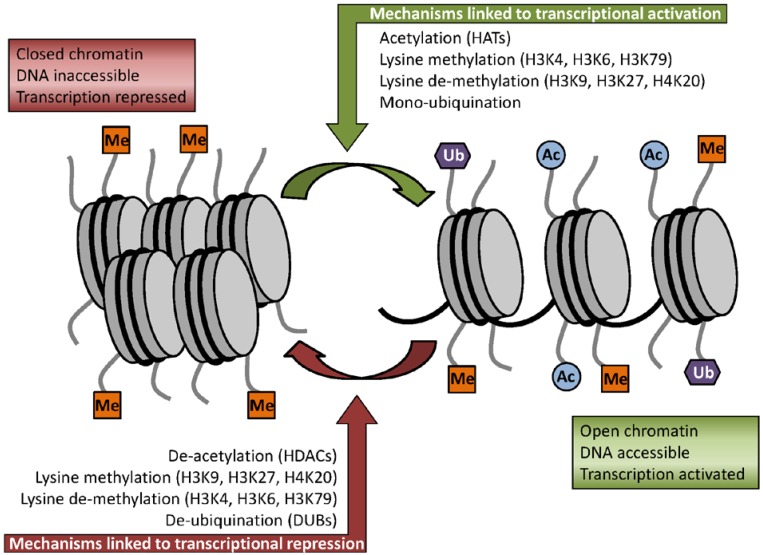
Some of the key histone modifications influencing gene expression. On the left is a representation of closed chromatin, in which the DNA is inaccessible to the transcriptional machinery and transcription is therefore repressed. Modifications to specific histone residues such as the addition of an acetyl group to a lysine residue (via a histone acetyl transferase, or HAT) lead to unfolding of the chromatin (as shown on the right hand side of the figure), which in turn allows the transcriptional machinery to access the DNA, resulting in transcriptional activation. Conversely, removal of this acetyl moiety (through a histone deacetylase, or HDAC) alters the histone configuration, once more returning the chromatin to the closed form. In the case of histone methylation, the effect on chromatin conformation depends on the specific lysine residue being methylated (as shown).

Two mechanisms for the function of histone modifications have been characterized; the relaxation/“unravelling” or compression of chromatin, and the recruitment of non-histone proteins. Depending on the type of modification and the amino acid residue involved, histone modifications can lead to either gene activation or repression. For example, addition of acetyl groups to the tail of histone H3 neutralizes the basic charge of the lysine and results in unfolding of the chromatin (Figure 1), thus allowing transcription to occur. Conversely, removal of these acetyl groups results in chromatin compression and prevents transcription [14]. These changes in chromatin structure can either encourage or exclude other proteins that can further modify the chromatin (e.g., remodelling ATPases). The steps involved in DNA transcription, replication and repair may each require a different set of modifications to remodel the chromatin and recruit the necessary enzymes involved [12].

**Table 1:** Types of histone modifications and the enzymes responsible (adapted from [12]).

Modification Type	Amino Acid Modified	Abbreviation	Examples of Modifying Enzymes Identified in Humans	Role
Acetylation	Lysine	K-ac	Histone Acetyltransferases (HATs): e.g., HAT1 Histone Deacetylases (HDACs): e.g., HDAC1	Transcription, Repair, Replication, Condensation
Methylation	Lysine Arginine	K-me1, -me2, -me3 R-me1, -me2	Lysine Methyltransferases: e.g., SUV39H1 Lysine Demethylases: e.g., LSD1/BHC110 Arginine Methyltransferases: e.g., PRMT4 Arginine Demethylases: e.g., JMJD6	Transcription, Repair Transcription
Phosphorylation	Serine Threonine	S-ph T-ph	Serine/Threonine Kinases: e,g. WEE1 Dephosphorylated by Phosphatases: e.g., PP4	Transcription, Repair, Condensation
Ubiquitination	Lysine	K-ub	Ubiquinases (Ubiquitin Ligases): e.g., RING1B Deubiquinating Enzymes: e.g., USP22	Transcription, Repair
SUMOylation	Lysine	K-su	Small Ubiquitin-like Modifier (SUMO) proteins: e.g., SUMO-1 De-SUMOylating Enzymes: Sentrin-Specific Proteases: e.g., SENP1	Transcription
ADP ribosylation	Glutamate	E-ar	ADP-Ribosyltransferases: e.g., ARTD1 (PARP1)	Transcription
Deimination	Arginine (to Citrulline)	K to Cit	Peptidylarginine Deiminases: e.g., PADI4	Transcription
Proline Isomerisation	Proline	P-*cis* to P-*trans*	Proline Isomerases: e.g., Pin1	Transcription

### 3.1. Acetylation

Histone acetylation is important in the regulation of gene expression and is normally associated with transcriptionally active gene dense regions, referred to as euchromatin [14]. Histone acetylation can be transient and must be maintained by enzymatic activity. Histone acetyltransferases (HATs) transfer the acetyl group (COCH_3_) from acetyl co-enzyme A to lysine residues (Figure 1). Conversely, HDACs remove the acetyl groups. Both HATs and HDACs are also able to modify a large variety of non-histone proteins whose activity depends on their acetylation status, such as transcription factors, chaperone proteins, signal transduction mediators, structural proteins, and inflammation mediators [14,15,16]. Consequently, changes in acetylation status have consequences for protein stability, protein-protein interactions, and protein-DNA interactions.

In general HATs can modify more than one lysine residue but some limited specificity has been detected [12]. Most acetyl groups are added to the *N*-terminal “histone tails”, which are more accessible for modification. However, a lysine within the core domain of H3 (K56) was found to be acetylated [17]; this K residue is facing toward the major groove of the DNA within the nucleosome and is therefore likely to affect histone-DNA interactions [12]. 

The reversal of acetylation by HDACs correlates with transcriptional repression. HDACs can regulate diverse cellular functions, including cell cycle progression, survival, and proliferation [18,19]. Four classes of HDACs have been identified in humans, comprising eighteen different proteins. Classes I, II, and IV belong to the RPD3/HDA1 family. Class I includes the constitutively expressed HDACs 1–3 and HDAC8. These are involved in cell proliferation and survival and are ubiquitously expressed. Class II HDACs seem to have tissue-specific roles and are subdivided into classes IIa (HDACs 4, 5, 7 and 9; which have weaker HDAC activity and can shuttle between the cytosol and the nucleus) and IIb (HDACs 6 and 10; which prefer non-histone proteins and are mostly found in the cytosol). Class IV consists solely of HDAC11. Classes I, II and IV are all zinc-dependent hydrolases and referred to as classical HDACs [20], while Class III consists of the NAD^+ ^dependent sirtuins (SIRT1–7), which are yeast Sir2 homologs [3,21]. Sirtuins have been implicated in influencing a wide range of cellular processes such as ageing, transcription, apoptosis, inflammation, axonal degeneration, cellular stress resistance, and metabolic regulation [22,23,24,25]. SIRTs 3–5 are specifically expressed in the mitochondria where they regulate the mitochondrial enzymes involved with energy production, metabolism, apoptosis, and intracellular signalling [26,27].

Because HDACs modulate a variety of cellular functions that are involved in carcinogenesis, cell growth, survival, and homologous recombination, they are considered a promising target for cancer therapy [18,28,29,30]. While HDACs are the particular focus of this review, we will also briefly describe the other key histone modifications. 

### 3.2. Methylation

Methylation of lysine or arginine residues may be one of three different forms: mono-, di- or trimethyl for lysines and either asymmetric or symmetric for arginines [12]. Lysine methylation is relatively stable and carried out by lysine methyltransferases, which have a high degree of specificity and usually only modify one single lysine on a single histone. This can result in either activation (H3K4, H3K36, and H3K79) or repression (H3K9, H3K27, and H4K20) of transcription [31]. As with lysine methylation, arginine methylation can activate or repress transcription. In contrast to the stability of lysine methylation, arginine methylation can be temporary and is carried out by arginine methyltransferases (e.g., PRMT4). Histone methylation is also a reversible process which involves the removal of methyl groups by demethylases specific for either methylated lysine [32] or arginine resides [33]. For example, the Jumonji domain-containing 6 protein (JMJD6) demethylates histone H3 at arginine 2 (H3R2) and histone H4 at arginine 3 (H4R3) [33]. However, unlike their lysine counterparts, very few arginine demethylases have been identified to date.

### 3.3. Ubiquitination

Ubiquitination (either mono- or poly-) is a very large modification found on histones H2A and H2B and is associated with either transcriptional repression or activation [34,35]. Because histones are the most abundant ubiquitinated proteins, this modification has critical roles in many processes in the nucleus, including transcription, maintenance of chromatin structure, and DNA repair. However, its specific functions are still less well understood than other histone modifications such as methylation and acetylation [36].

Like most other histone modifications, monoubiquitination of histones H2A and H2B is reversible by ubiquitin-specific peptidases known as deubiquitinating enzymes (DUBs). Besides mono-ubiquitination, histones H2A and H2B can also be modified by ubiquitin chains (polyubiquitination; [36]). Mono-ubiquitination is likely to recruit additional factors to chromatin and may keep chromatin open by a “wedging” process [12] whereas polyubiquitination is required for the DNA repair response and targeted proteasome-mediated degradation, respectively (as reviewed in [36]).

### 3.4. SUMOylation

SUMOylation is also a very large modification that has been shown to take place on all four core histones; antagonising both acetylation and ubiquitylation, which occur on the same lysine residue. In humans, there are four small ubiquitin-like modifier (SUMO) isoforms (SUMO-1 to -4) encoded by different genes. Although SUMO proteins are associated with transcriptional regulation, recent work using HeLa cells showed that a relatively high percentage of the most active genes (49%) had their promoters modified with bound SUMO-1 [37]. SUMOylation of chromatin-associated factors has also been associated with stimulation of transcription [37]. Typically, only a small fraction of a given protein is SUMOylated and this modification is rapidly reversed by the action of deSUMOylating enzymes belonging to the sentrin-specific protease (SENP) family [38].

### 3.5. Other Modifications

*Phosphorylation*: Histone phosphorylation is best known for its role during cellular response to DNA damage [39]. However, it also has crucial roles in chromatin remodelling linked to other nuclear processes such as transcription regulation, and chromatin condensation associated with mitosis, meiosis and apoptosis [40]. Histone phosphorylation can occur on serine, threonine and tyrosine residues. All four nucleosomal histone tails contain acceptor sites that can be phosphorylated by a number of protein kinases and dephosphorylated by phosphatases [41].

*Deimination*: An alternative pathway for the reversal of arginine methylation involves the conversion of an arginine in either histone H3 or H4 to a citrulline [42]. This is termed deimination because the methyl group is removed along with the imine group of arginine and is catalysed by peptidylarginine deiminase 4 (PADI4; also known as PAD4) [12]. Deimination can change the structure and function of histone proteins due to increased hydrophobicity of the protein, which results in protein folding changes. Since conversion to citrulline prevents arginines from being methylated, this has the potential to control gene expression by competing with transcriptional activation by arginine methylation [43,44]. Converting citrulline back to arginine has not yet been described [12].

*ADP Ribosylation*: Unlike other histone modifications, ADP ribosylation is much less studied with respect to function. All five histone proteins can be either mono- or poly-ADP-ribosylated. ADP ribosylation involves the transfer of ADP-ribose from nicotinamide adenine dinucleotide (NAD^+^) by ADP-ribosyltransferases (ARTs; e.g., ARTD1 (PARP1)) which are subsequently removed by PARPs (poly-ADP-ribose polymerases) [45]. Many cellular processes such as the genotoxic stress response, cell cycle regulation, gene expression, differentiation, and ageing are thought to be influenced by this modification [46].

*Proline Isomerization*: Prolines exist in either a *cis* or *trans* conformation, which serves as a regulatory switch in signalling pathways [47]. These conformational changes can severely distort the polypeptide backbone. Most of the early work in this area centred on a proline isomerase found in yeast (Fpr4) which catalyses the isomerization of proline P30 and P38 in histone H3 [47]. Here, proline isomerization was shown to be a novel noncovalent histone modification that regulates transcription and is necessary for histone lysine methylation. Recently, phosphorylation-dependent proline isomerization undertaken by Pin1, a phosphorylation-specific prolyl isomerase present in the nucleus, was shown to be a chromatin regulatory mechanism that promotes a more compact chromatin state in histone H1 [48].

## 4. HDACs in Health and Disease

Dysfunction of HDAC enzymes has been linked with a variety of human diseases, as shown in Table 2. In general, the class I enzymes appear to have a role in the survival and proliferation of cancer cells, while class II, notably HDAC 8, may be responsible for tumorigenesis [49]. HDACs are implicated in cancer partly through their silencing of tumour suppressor genes, e.g., the hypoacetylation status of the *p21^WAF1^* promoter and its corresponding gene inactivation can be reversed by HDAC inhibitors via histone hyperacetylation of in the promoter [50]. Here, we briefly describe the role of each class of HDAC in health and disease. 

### 4.1. Class I HDACs (HDACs 1–3 and HDAC8)

Because these are ubiquitously expressed and involved in cell proliferation and survival, aberrations in their gene expression have been implicated in a wide range of cancers. *HDAC1*–*HDAC3* genes are over-expressed in ovarian cancer tissues and probably have a significant role in ovarian carcinogenesis [51]; these HDAC isoforms are also highly expressed in Hodgkin’s Lymphoma (HL). However, decreased *HDAC1* expression is accompanied by worse outcome in HL [52]. Over-expression of *HDAC1* has also been reported in prostate and gastric cancers [53,54], while contrastingly, under-expression was reported in colorectal cancer [55]. 

Although changes in *HDAC2* expression have also been identified in a number of cancers, an inactivating frameshift mutation appears to leave cells more resistant to HDAC inhibitors (HDACi) [56]. These findings suggest that the *HDAC2* mutational status of patients should be assessed before using HDACi therapies to treat certain cancers. A reduction in HDAC2 protein activity and expression were observed in the lungs of patients with chronic obstructive pulmonary disease. In patients with very severe disease, there was a >95% reduction in the expression of HDAC2 [57]. This may account for the increased inflammation and corticosteroid resistance observed in these patients. However, *HDAC3* was shown to be overexpressed in lung cancers [58] reinforcing the point that it is not a “one size fits all” approach to using HDACi therapies to treat diseases where HDACs have been implicated. 

In terms of contributing to healthy cellular responses, *HDAC2* was found to be a key regulator of diabetes in mice [59]. In contrast, variants of *HDAC3* contribute to an increased prevalence of type 2 diabetes mellitus (DM) in the Chinese Han population [60]. This supports previous work which showed that mice with a liver-specific deletion of HDAC3 have severe hepatosteatosis and, notably, increased insulin sensitivity without changes in insulin signalling or body weight [61]. *HDAC3* (along with *HDAC4*) also appears to have a role in long-term memory. Homozygous deletions of *HDAC3* in mice resulted in improved long-term memory [62] while deletion of *hda4*, a homologue of *HDAC4* in a *Caenorhabditis elegans* model, resulted in enhanced learning and long-term memory [63]. Mutations in the *HDAC3* and *HDAC4* genes have also been associated with the pathophysiology of schizophrenia [64].

Classical HDACs have a major role in epigenetic gene silencing during development. *HDAC8* specifically controls the patterning of the skull. Mutations in the *HDAC8* gene are associated with Cornelia de Lange disease; a syndromic form of intellectual disability characterized by facial dysmorphisms [65]. Knockdown of *HDAC8* by RNA interference was shown to inhibit growth of human lung, colon, and cervical cancer cell lines, highlighting the importance of this HDAC in tumour cell proliferation [20].

### 4.2. Class IIa HDACs (HDACs 4, 5, 7 and 9)

These appear to have tissue-specific roles and can shuttle between the cytosol and the nucleus. HDAC4 acts as a transcriptional repressor, so it is unsurprising that *HDAC4* mutations have been identified at significant frequency in breast and colorectal cancers [66]. *HDAC4* was shown to be down-regulated in colon and lung tumours whereas no significant differences were observed in prostate carcinomas, implying that there may be a novel set of regulatory suppressor genes involved in human colon and lung tumours [67]. However, these results are in contrast to those of Halkidou [68], who found nuclear accumulation and increased expression of *HDAC4* in hormone refractory (HR) cancer of the prostate which coincided with the loss of androgen sensitivity. When termed “HR”, prostate cancer can no longer be cured by conventional therapy. The differences observed between the two studies are most likely due to changes in *HDAC4* expression in response to the development of the aggressive phenotype of late stage prostate cancer.

An *HDAC4* polymorphism, at rs12477314, was also found to affect foetal, childhood and adult lung function [69]. *HDAC4* haploinsufficiency has been implicated in Brachydactyly-Mental Retardation (BDMR) syndrome and the psychomotor and behavioural abnormalities observed in these patients; particularly the syndrome-specific facial dysmorphism pattern [70,71]. *HDAC4* is also associated with Huntington’s Disease (HD). Using *HDAC4* knock-down mouse models of HD, Mielcarek *et al.* [72] showed that neuronal and cortico-striatial synaptic function was restored. This was accompanied by an improvement in motor coordination, neurological phenotypes, and increased lifespan.

Anorexia nervosa and bulimia nervosa are common and severe eating disorders of unknown aetiology. Work by Cui *et al.* [73] identified a missense mutation in the *HDAC4* gene that segregated with illness. HDAC4 was shown to interact with the oestrogen-related receptor α (ESRRA) gene and strongly repress the expression of known ESRRA-induced target genes. Overall, their findings suggested that mutations in HDAC4 result in decreased ESRRA activity, and this in turn increases the risk of developing eating disorders [73].

Decreased expression of the *HDAC5* gene was also observed in colorectal cancer while overexpression of *HDAC7* has been reported in this disease [55]. HDAC5 has also been shown to be involved in major depression. Levels of *HDAC5* mRNA in leukocytes were significantly higher in drug-free depressive patients than control patients and these mRNA levels dropped to levels observed in control patients after 8-weeks of drug treatment. This suggests a role for *HDAC5* expression in the systemic pathophysiology of major depression [74].

*HDAC7* was found to be significantly associated with pancreatic adenocarcinomas, and this increased expression of *HDAC7* discriminates pancreatic adenocarcinomas from other types of pancreatic tumours [75]. In contrast, *HDAC9* has a key role in the development and differentiation of many types of cells, including regulatory T cells. Systemic autoimmune diseases such as lupus, diabetes, and rheumatoid arthritis have dysfunctional effector T cells; this suggests that HDAC9 may act as an epigenetic switch in effector T cell-mediated systemic autoimmunity [76]. Disruptions in the *HDAC9* gene have been implicated in Peters’ anomaly; a congenital defect of the anterior chamber of the eye [77]. Genetic variability in *HDAC9*, along with variants in *HDAC11*, *SIRT4* and *SIRT5,* has also been shown to influence brain volume in multiple sclerosis (MS) patients, as assessed used neuroimaging methods [78].

### 4.3. Class IIb HDACs (HDAC6 and 10)

These prefer non-histone proteins and are mostly found in the cytosol. HDAC6 has been shown to have a central role in protein aggregate elimination and has been implicated in a number of neurodegenerative diseases such as Alzheimer’s, Parkinson’s, and Huntington’s [3]. The expression of *HDAC6* increases significantly in the hippocampus and other relevant brain regions in patients with Alzheimer’s disease (AD). However, it is still unknown when and how *HDAC6* expression increases during disease progression. There is growing evidence to suggest that increased *HDAC6* expression contributes to AD-associated neurodegeneration, although beneficial effects have also been identified implying that *HDAC6* may be a logical therapeutic target for AD [79].

HDAC6 also has a role in neuronal oxidative stress and mitochondrial transport, and plays an important part in both cell cycle regulation and ciliary disassembly. Polycystic liver disease (PLD) is a member of the cholangiopathies, a group of liver diseases in which cholangiocytes (the epithelia lining of the biliary tree) are the target cells. These diseases are caused by mutations in genes involved in intracellular signalling pathways, cell cycle regulation, and ciliogenesis. HDAC6 protein expression was shown to increase 6-fold in human PLD and its pharmacological inhibition reduced cholangiocyte proliferation and cyst growth. This suggests that HDAC6 may represent a potential novel therapeutic target for cases of PLD [80]. However, it may not be an important therapeutic target in selected lymphoid malignancies; *HDAC6* was rarely expressed (compared with *HDACs* *5*, *8*, and *10*) in primary lymphoma cases [81]. Although there is very little in the literature with regards to *HDAC10*, this gene (along with most of the other *HDAC* genes) has been shown to be overexpressed in Chronic Lymphocytic Leukaemia [82].

### 4.4. Class III HDACs (SIRT1–7)

These have a critical role in a wide range of cellular processes such as ageing, transcription, cell survival, DNA repair, apoptosis, and inflammation. Sirtuins appear to have contradictory roles in disease; on the one hand they control many vital functions involved in cellular protection but on the other hand, they are also involved in several pathologies such as metabolic diseases, neurodegenerative disorders, and cancer [83,84]. The most studied of these, *SIRT1*, has been shown to be significantly up-regulated in different types of cancer including acute myeloid leukaemia (AML), prostate, colon, and skin cancers [84]. However, it also seems to have contradictory roles as either a tumour suppressor or tumour promoter [83,85]. *SIRT1* was shown to be over-expressed in breast, colorectal and prostate cancer cell lines [86] but under-expressed in another study which looked at its expression in tissue from patients with colorectal cancer [55]. Both *SIRT1* and *SIRT6* were shown to be over-expressed in patients with Chronic Lymphocytic Leukaemia [82].

Sirtuins also have a vital role in neuroprotection and while the expression of some *SIRT* genes is decreased in many neurodegenerative diseases, others are increased. For example, a recent study of patients in different stages of neurofibrillary degeneration showed that expression of *SIRT1* is decreased and redistributed in neuron cells during AD progression which suggests stepwise loss of neuroprotection. However, in contrast to *SIRT1*, the expression of *SIRT5* increases during the progression of AD [87]. In fact, SIRT5 has only recently been discovered to possess broader deacylase activity, and functions primarily as a lysine demalonylase and desuccinylase [88,89]. However, the significance of these post-translational modifications is still unclear [90]. *SIRT2* has been associated with susceptibility to AD. Wei *et al.* [91] showed that a SNP in the *SIRT2* gene was associated with human AD risk. There is also growing evidence that posttranslational modifications of histone proteins are involved with several critical processes relevant to multiple sclerosis (MS) and are sensitive to environmental factors previously implicated in MS (e.g., nutritional status and vitamin B3) [92,93]. Understanding how genetic variants influence these processes is an important challenge. Recently, Inkster *et al.* [78] showed that individual genetic variants of the *SIRT4* and *5* genes, as well as *HDACs 9* and *11*, were linked to brain volume in MS patients. 

*SIRT3* has been implicated in tumorigenesis. Its reduction in several cancers leads to an increase in reactive oxygen species (ROS) production, which results in enhanced tumour growth [94]. A recent study by Yang *et al.* [94] provided the first evidence that both *SIRT3* gene and protein expression are significantly decreased in gastric cancer patients, suggesting that *SIRT3* may have a role as a mitochondrial tumour suppressor in this disease via its direct control of HIF-1α. Recently, roles for *SIRT6* and *SIRT7* in tumorigenesis have also been suggested. *SIRT6* may be involved as a result of its control of the NFκB pathway and DNA double-strand repair. *SIRT7*, whose expression inversely correlates with the tumourigenic potential in several murine cell lines [95], displays increased expression levels in breast cancer compared to healthy tissue [96]. Differential sirtuin expression between non-malignant and malignant breast tissue may thus have potential diagnostic and therapeutic implications.

### 4.5. Class IV HDACs (HDAC11)

*HDAC11* is primarily expressed in heart, smooth muscle, kidney, and brain tissues. Gloghini *et al.* [81] were the first group to report on the pattern of class IV *HDAC11* expression in lymphoma where they found that *HDAC11* was expressed in all lymphoid cell lines. Interestingly, *HDAC11* was expressed in primary non-Hodgkin’s Lymphoma cases but not in Hodgkin’s Lymphoma cases [81]. As with the *SIRT4* and *SIRT5* genes, variants in *HDAC11* were also shown to influence multiple sclerosis in terms of brain volume [78].

**Table 2:** Effect of HDAC dysregulation on human disease (adapted from [97]).

	Name	Type of dysregulation	Disease Implicated	Reference(s)
**Class I**	HDAC1	*HDAC1* overexpression	Prostate cancer Gastric cancer Ovarian cancer Hodgkin’s lymphoma	[53,68] [54,98] [51] [52]
		*HDAC1* underexpression	Colorectal cancer	[55]
	HDAC2	Truncating *HDAC2* mutation	Colonic, gastric and endometrial cancers	[55,56]
		*HDAC2* overexpression	Ovarian cancer Hodgkin’s lymphoma	[51] [52]
		Reduction in activity and expression	Chronic obstructive pulmonary disease	[57]
	HDAC3	SNP variants	Type 2 diabetes Schizophrenia	[60] [64]
		Liver-specific deletion	Severe hepatosteatosis and increase in insulin sensitivity	[61]
		*HDAC3* overexpression	Ovarian cancer	[51]
		Increased HDAC3 protein expression	Hodgkin’s lymphoma Colon cancer	[52] [99]
	HDAC8	*HDAC8* mutations	Cornelia de Lange disease	[65]
**Class IIa**	HDAC4	Splice-site/missense mutations	Breast cancer Eating disorders	[66] [73]
		SNP variant	Lung function Schizophrenia	[69] [64]
		*HDAC4* overexpression	Prostate cancer Colon cancer Lung cancer Breast cancer	[68] [67] [67] [55]
		Haploinsufficiency	Pyschomotor and behavioural abnormalities	[70,71]
		Reduction	Huntington’s disease	[72]
	HDAC5	*HDAC5* underexpression *HDAC5* overexpression	Colorectal cancer Major depression	[55] [74]
	HDAC7	Over expression	Colorectal cancer Pancreatic cancer	[55] [75]
	HDAC9	Gene variants Gene disruption	Multiple sclerosis Peters’ anomaly	[78] [77]
**Class IIb**	HDAC6	*HDAC6* overexpression	Neurodegenerative diseases (e.g., Alzheimer’s disease)	[3,79]
		Increased HDAC6 protein expression	Polycystic liver disease	[80]
		X-linked *HDAC6* mutation	Adult-onset Alexander disease	[100]
		Little or no *HDAC6* expression	Hodgkin’s lymphoma	[81]
	HDAC10	*HDAC10* overexpression	Chronic lymphocytic leukemia	[82]
**Class III**	SIRT1	Overexpression	Breast, colorectal and prostate cancer	[86]
		*SIRT1* underexpression	Colorectal cancer	[55]
	SIRT2	Polymorphism	Alzheimer’s disease	[91]
	SIRT3	mRNA and protein underexpression	Gastric cancer	[94]
	SIRT4	Gene variants	Multiple sclerosis	[78]
	SIRT5	Gene variants *SIRT5* overexpression	Multiple sclerosis Alzheimer’s disease	[78] [87]
	SIRT6	Decreased *SIRT6* expression	Liver cancer and cirrhotic livers	[101]
	SIRT7	*SIRT7* overexpression	Breast cancer	[96]
**Class IV**	HDAC11	Gene variants	Multiple sclerosis	[78]

## 5. HDAC Inhibitors

Given that altered or aberrant HDAC activity has been associated with a wide variety of diseases, compounds which are able to inhibit HDAC activity offer potential solutions to prevent or ameliorate these. Interestingly, compounds which could inhibit histone deacetylation activity were discovered before the isolation of an enzyme with HDAC activity. The first significant HDAC inhibitor to be identified was *n*-butyrate, which caused a reversible accumulation of hyperacetylated histones within cell nuclei [102]. More than a decade after this discovery, trichostatin A (TSA) and trapoxin A (TPX) were also shown to be potent inhibitors of HDAC activity [103,104]. TSA was found to be a reversible inhibitor of HDACs, whereas TPX, first isolated from fungal metabolites, is an irreversible inhibitor [105]. The first human HDAC was discovered and cloned by the Schreiber laboratory using the HDAC inhibitor TPX to isolate bound HDAC molecules [106].

HDAC inhibitors (HDACi) belong to a large and diverse family that include a range of naturally occurring as well as synthetic compounds. Given their structural diversity, HDACi also differ in terms of function and specificity. Some HDACi, such as TSA, are pan-HDAC inhibitors while others are class-selective or isoform-selective inhibitors. This means individual HDACi are able to exhibit a range of effects, such as growth arrest, cell differentiation, and apoptosis in malignant cells, which may be specific to certain cell types [107]. HDACi can be grouped into at least four major classes, in order of decreasing potency: hydroxamic acids/hydroxamates (e.g., TSA); cyclic peptides (e.g., Romidepsin; FK-228); benzamides (e.g., pimelic diphenylamide; 106); and aliphatic acids (e.g., valproic acid) [5,6]. Class III HDACs (sirtuins) are dependent on NAD^+^ and are therefore inhibited by nicotinamide as well as derivatives of NAD, dihydrocoumarin, napthopyranone and 2-hydroxynaphaldehydes [5].

Because a number of proteins and transcription factors other than histones are also modified by acetylation, there are a wide range of biological effects caused by HDAC inhibition which remain unknown. However, the main result of HDAC inhibition is hyperacetylation and, consequently, gene expression. Apart from the effects observed on gene transcription, evidence is accumulating to show that HDAC inhibitors influence chromatin stability, mitosis, and DNA repair mechanisms [108]. Because increased HDAC activity and expression is common in the development of many cancers, HDACi have shown promise as potential cancer treatments, either on their own or in combination with other therapies such as chemotherapy drugs [109]. HDACi were shown to induce apoptosis in a wide range of malignant cell lines and increase expression of *p21* [50]. They have also been shown to ameliorate chemically-induced colitis in mice [110] and show potential for the treatment of cardiac, neurodegenerative and inflammatory lung disease [111,112,113]. Most importantly, normal cells appear relatively resistant to the effects of HDACi, thereby reducing any negative side effects [49,109].

### 5.1. Naturally Occurring HDAC Inhibitors

Many of the first HDACi to be discovered were naturally occurring microbial metabolites such as TSA (isolated from *Streptomyces hygroscopicus*) and the depsipeptide FK228 (isolated from *Chromobacterium violaceum*). While TSA is a hydroxamic acid-based pan-HDAC inhibitor, FK228 is a natural cyclic peptide which selectively inhibits HDACs 1 and 2. TSA remains one of the most potent HDAC inhibitors available. However, its development as a therapeutic agent has been neglected due, in part, to production costs. TSA has been shown to affect gene expression in tumour cells as well being as a profound therapeutic agent in several other diseases such as asthma [104,111,114].

Other naturally occurring HDACi have been isolated from fungi (e.g., depudecin and trapoxin A and B) and marine life (e.g., largazole and azumamides). The HDAC activity of these novel marine natural products can occur at nanomolar concentrations [49,115,116]. Given their rich source of biologically active compounds, extensive efforts have also been undertaken to identify novel plant-derived HDACi. Allyl derivatives from garlic were among the first compounds described to impact histone acetylation status [108]. Other well-characterized plant HDACi include sulforaphane (SFN) isolated from cruciferous vegetables, and quercetin, found in a variety of fruit. Molecular modelling studies with other dietary compounds, such as biotin, vitamin E metabolites and α-lipoic acid suggest that these may also have potential as HDACi [20]. Given the promise of HDACi to treat a variety of diseases, particularly cancer, there is growing interest in the potential of dietary compounds that possess HDAC inhibition activity. Dietary HDACi are discussed in more detail below (see Section 6).

### 5.2. Synthetic HDAC Inhibitors

Natural HDAC inhibitors, such as TSA, have been shown to inhibit HDACs primarily through chelation of the zinc in the catalytic pocket. Consistent with the structure of the enzyme-inhibitor complex, most of the HDAC inhibitors consist of cap, spacer, and functional groups. Many of the new synthetically-derived HDAC inhibitors have been designed based on structural information obtained from naturally occurring compounds. Currently, the most advanced HDAC inhibitor is a compound called SAHA (suberoylanilide hydroxamic acid; also known as vorinostat or Zolinza^®^; Merck, Darmstadt, Germany) which was discovered through extensive surveys of small polar molecules capable of inhibiting HDAC enzymes [117]. SAHA is a hydroxamic acid-based pan-HDAC inhibitor. Many of these synthetically-derived HDACi have been recently reviewed so will not be described here [49,109].

### 5.3. Use of HDAC Inhibitors in Treating Disease

In addition to suberoylanilide hydroxamic acid (SAHA), and Romidepsin (FK228), approved by the U.S. Food and Drug Administration (FDA) for the treatment of cutaneous T-cell lymphoma in 2006 and 2009, respectively, there are at least 15 different HDAC inhibitors currently undergoing clinical trials in patients with a wide range of different cancers either as monotherapy or in combination therapy [107,109]. HDACi also have a long history of use in psychiatry and neurology as mood stabilizers and anti-epileptics, e.g., valproic acid [118].

## 6. Dietary HDAC Inhibitors as Therapeutics

Diet-related factors are thought to account for approximately 30% of cancers in developed countries [119]. Lifestyle and diet have been identified as major risk factors in a number of diseases such as prostate, gastric, colorectal, and lung cancers [120,121,122]. Strong evidence is emerging that vegetables, fruits, whole grains, dietary fibre, certain micronutrients, and specific fatty acids protect against some cancers and other diseases by inhibiting HDAC activity, consistent with an increased appreciation of the importance of dietary factors in health and well-being.

HDAC inhibitors represent a promising therapeutic approach for a wide range of disorders including inflammatory diseases and cancer. Dietary compounds that can alter histone acetylation therefore offer novel strategies for preventing, delaying or reversing these diseases through simple nutritional choices. Ease of accessibility, as well as their ability to act as primary protective agents, has meant that manipulation of histone structure and function via consumption of specific nutrients and other compounds has been gaining increasing interest [16]. For instance, in the case of inflammatory bowel diseases (IBD), particular food constituents may be important for modulating specific epigenetic changes to reduce inflammation and ultimately lower the risk of colon cancer; all by making subtle modifications to the histone code [14]. Many of the dietary compounds that have been identified as having HDACi activity are listed in Table 3.

A growing number of the dietary HDACi reported in the literature are generated as metabolites during the course of digestion. For example, butyrate, a short-chain fatty acid generated via the fermentation of dietary fibre by the colonic microbiota, was shown over 35 years ago to affect histone status in HeLa and erythroleukaemia cells, via competitive HDAC inhibition [102]. The daily consumption of dietary fibre, such as cereal, may therefore be important in the maintenance of good health and protection against cancer, cardiovascular disease and Type 2 Diabetes [108,123]. Natural organoselenium compounds, such as *Se*-methylselenocysteine (MSC), are found in selenium-rich foods such as Brazil nuts. During metabolism, glutamine transaminase K converts MSC to methylselenopyruvate (MSP) which was found to be a potent HDAC inhibitor in prostate and colon cancer cells [124,125]. A more in-depth explanation of organoselenium metabolism can be found in Pinto *et al.* [126]. More recently, Cao *et al.* showed that MSC provides selective protection against the side effects induced by chemotherapy drugs (e.g., diarrhoea, stomatitis, alopecia) and enhances further anti-tumour activity, thus resulting in improved cancer therapy [127].

Digestion of *Allium* vegetables (e.g., garlic and onions) produces seleno-α-keto acid metabolites which have also been shown to inhibit HDAC activity [108]. These vegetables also contain a number of organosulphur compounds which can be converted into thiols (e.g., allyl mercaptan) which also have HDACi activity. Likewise, biologically active isothiocyanates such as SFN are produced when cruciferous vegetables such as broccoli are chewed. These are metabolized via the mercapturic acid pathway to generate a number of intermediates with HDACi activity. SFN has been shown to inhibit HDAC activity in human colon cancer cells accompanied by both global and localized histone hyperacetylation, G_2_/M cell cycle arrest, and increased apoptosis [128]. Another dietary compound formed by the metabolism of cruciferous vegetables (including broccoli, cabbage, Brussels sprouts, cauliflower, and kale) is 3,3′-di-indolylmethane (DIM) [129]. The anticancer activity of DIM has been demonstrated in both breast and colon cancer cells [130].

Bitter melon (*Momordica charantia*) is a plant that is both consumed as a vegetable and used medicinally. It grows in tropical areas worldwide and contains a protein called MCP30 which has been shown to demonstrate inhibition of HDAC1 in prostate cancer cells [131]. Parthenolide is a sesquiterpene lactone found in highest concentration in the flowers and fruit of feverfew (*Tanacetum parthenium*). It is used in a number of herbal remedies although it can be eaten in the plant form. Parthenolide was found to specifically deplete HDAC1 protein levels without affecting other class I/II HDACs [132].

**Table 3:** Examples of dietary compounds identified as inhibiting HDAC activity.

Dietary Component	Food Source	References
Allicin	Garlic	[133]
*Bis*-(4-hydroxybenzyl)sulfide	Polygonaceae *(Pleuropterus ciliinervis* Nakai*)*	[134]
Caffeic acid	Intestinal metabolite of nutritional polyphenols	[135]
Catechins (e.g., green tea polyphenols)	Tea (*Camellia sinensis*), particularly green tea	[136]
Coumaric/hydroxycinnamic acid	Cinnamon	[135]
Curcumin	Turmeric	[137]
Diallyl disulfide	Garlic	[138,139]
3,^3^ʹ-di-indolylmethane	Cruciferous vegetables, e.g., broccoli	[129]
Equol	Soy	[140]
Flavonoids, e.g., Apigenin Chrysin	Common fruits and vegetables, e.g., grapefruit, parsley and chamomile Fruits, vegetables, olive oil and red wine	[141] [142]
Genistein	Soy	[140,143]
Isothiocyanates (e.g., sulforaphane)	Cruciferous vegetables, e.g., broccoli	[144,145]
MCP30	Bitter melon	[131]
Organoselenium compounds e.g., Se-methyl-l-selenocysteine	Broccoli, Garlic, Onion	[125]
Parthenolide	Feverfew (*Tanacetum parthenium*)	[132]
Pomiferin	Osage orange/Hedge apple (*Maclura pomifera*)	[146]
Quercetin	Citrus, apple, berries	[147]
Resveratrol	Grapes, wine, eucalyptus	[148,149]
Selenium compounds	Brazil nuts	[124,127]
Sesquiterpenoids	Shampoo Ginger (*Zingiber zerumbet*)	[150]
Ursolic acid	Basil	[151]

There is a growing number of dietary plant flavonoids identified as having HDACi activity, which is not surprising given their purported anti-cancer and anti-inflammatory properties (as reviewed in Yao *et al.* [122]). For example, apigenin and chrysin are both found in a wide range of fruit and vegetables and have been shown to induce growth arrest via inhibition of HDACs in prostate cancer and melanoma cells, respectively [141,142,152].

A small number of dietary compounds appear to have dual effects on histone acetylation status. Resveratrol, a naturally occurring compound found in grapes, wine and eucalyptus, is a potent activator of sirtuins (Class III HDACs) and in particular, SIRT1 [148,149]. Resveratrol has also been shown to inhibit the other eleven human HDACs (Class I, II and IV) in a dose-dependent manner [153]. Curcumin [154,155] and genistein [140,143] exhibit both HDAC inhibitor and HAT activator activities. Curcumin has been shown to reduce HDAC4 expression and induce apoptosis and cell cycle arrest at the G_2_/M phase in medulloblastoma cells, both *in vitro* and *in vivo*. As medulloblastoma is the most common brain tumour found in children, curcumin may offer a dietary means to prevent this disease.

Quercetin, a ubiquitous dietary flavonoid found in many fruits, behaves as a HAT inhibitor and SIRT1 activator [156] but also inhibits HDAC1 and DNMT1 (a DNA methyl transferase) in tumour cells [147]. Thus regular consumption of foods rich in this compound may prevent or ameliorate certain cancers and purified quercetin may act as an ideal candidate for multi-targeted cancer prevention and therapy [147].

Dietary plant polyphenols are a rich untapped source of potential new HDACi. Plants produce a vast range of secondary metabolites, many of which have yet to be characterized. Extensive research has been undertaken to understand the mechanisms underlying the beneficial effects of consuming green tea. Recent work by Thakur *et al*. [136] demonstrated that green tea polyphenols, of which the major constituent is epigallocatechin-3-gallate, can inhibit class I HDACs to induce cell cycle arrest and apoptosis. The continued development of high throughput HDACi assays will help to identify novel plant polyphenols which have HDACi activity and further elucidate the particular HDACs they restrict [115].

Overall there is very little in the literature with regards to the effect of whole foods on HDAC activity and histone acetylation [157]. Healthy human volunteers fed broccoli sprouts (1 cup/day; 68 g), which equated to a daily dietary intake of 105 mg of the HDAC inhibitor SFN, showed hyperacetylation of histones H3 and H4 in normal circulating blood cells. The level of HDAC inhibition was greater than, or equal to, that achieved with the clinical HDAC inhibitor vorinostat [158]. While the evidence gathered from *in vitro* studies has made significant contributions to the understanding of the epigenetic mechanisms associated with dietary agents, additional clinical work is still required to examine the safety profiles of various doses of these compounds together with the possible interactions between different food components [159]. An important consideration is whether or not the concentrations of these dietary compounds required for inhibition of HDAC activity are achievable under normal physiological conditions or whether it is more feasible to extract these compounds from natural sources and use them to fortify existing foods or beverages.

The reality is that many of the recently identified dietary HDACi are essentially used as templates to create the next generation of synthetic HDACi, rather than encouraging people to consume more fruit and vegetables. Rajendran *et al.* [157] suggest that the toxicity and drug resistance observed with some clinically used HDACi could be avoided by lowering the dose of these, while supplementing with dietary HDACi in the form of broccoli sprouts or other foods, which must then be metabolized to their active forms. This may provide for a more sustained level of HDAC inhibition that the “fast-on/fast-off” agents currently in clinical use [157].

### Effect of Cooking on HDACi Activity

While the preparation of vegetables for consumption varies widely, they are often cooked before being eaten. Several studies have looked at the effect of different cooking methods on the bio-availability and content of nutrient and health-promoting compounds in vegetables, particularly in broccoli which contains high levels of chemopreventive compounds such as the glucosinolates [160,161,162]. One recent study of domestic cooking methods showed that of all the cooking treatments (steaming, microwaving, boiling, stir-frying and stir-frying followed by boiling—both traditional methods used in Chinese cooking), steaming resulted in the best retention of nutrients in broccoli and lowest loss of glucosinolates, whereas the other methods caused significant losses of chlorophyll, vitamin C, total soluble protein, and soluble sugars [162]

## 7. Conclusions and Future Directions

It is now well established that histones have an important dynamic role in the regulation of gene expression, via the wide range of histone modifications which are known to exist. It is also clear that gene regulation through such means is intricately linked to the appropriate function of an organism, and thus to its overall health status. Therefore, it follows that a better understanding of the mechanisms by which these modifications occur, and how the various intrinsic and extrinsic factors which act upon these mechanisms may be influencing gene regulation, has important consequences for human health.

In this review, we have focused on histone acetylation as an example of such a modification, with a particular emphasis on the dietary factors that influence it. This is primarily because there is an increasing understanding of the functional components of foods, and how our diet can have important roles that go beyond the provision of nutrients. These functions include signalling, acting as key substrates in biochemical pathways, and anti-oxidant activity. We have described one example of such a class of functional food compound, dietary HDAC inhibitors, which specifically act to influence histone acetylation.

We have described in some detail, although by no means exhaustively, the range of disorders which are linked to HDAC dysfunction. The number and variety of these disorders serve to emphasize the importance of the appropriate regulation of gene expression through modification of histone residues in the maintenance of health and well-being. There is clearly a growing interest in the therapeutic use of HDAC inhibitors in the treatment of several disorders linked to HDAC dysfunction, and this is likely to continue for the foreseeable future. However, there are potential side-effects for a number of these, and this, combined with the growing interest in the maintenance of health rather than the treatment of disease, means that improved characterization of dietary HDAC inhibitors provides an opportunity to generate positive health outcomes through a plausible mechanism within the context of a whole food or diet.

In summary, HDAC inhibition is one mechanism by which gene expression is appropriately regulated, with important implications for health. A better understanding of dietary HDACi should yield significant benefits in terms of maintaining human health through appropriate dietary choices, whereas the continued development of therapeutic HDACi (either from dietary sources, or synthetically) represents a complementary approach at a point further along the disease continuum.
